# Beekeepers as guardians of apitherapeutic knowledge in Estonia, SW Ukraine, and NE Italy

**DOI:** 10.1186/s13002-025-00764-6

**Published:** 2025-03-10

**Authors:** Raivo Kalle, Nataliya Stryamets, Denisa Lorena Cutuca, Julia Prakofjewa, Edy Fantinato, Ingvar Svanberg, Giulia Mattalia, Renata Sõukand

**Affiliations:** 1 Estonian Literary Museum, Tartu, Estonia; 2 Roztochya Nature Reserve, Ivano-Frankove, Ukraine; 3 Faculty of Forest Sciences, School for Forest Management, Swedish University of Agricultural Sciences, Skinnskatteberg, Sweden; 4 Department of Environmental Sciences, Informatics and Statistics, Ca’ Foscari University of Venice, Venice, Italy; 5 Institute for Russian and Eurasian Studies, Uppsala University, Uppsala, Sweden; 6 Institut de Ciència i Tecnologia Ambientals, Universitat Autònoma de Barcelona (ICTA- UAB), Cerdanyola del Vallès, Bellaterra, 08193 Spain

**Keywords:** Alternative and complementary medicine, Bee therapy, Folk remedies, Honey, Pollen, Propolis

## Abstract

**Background:**

Bees have been important to people in Europe in many ways. Honey was the only sweetener available for a long time. The introduction of frame hives allowed for the collection of various hive products and better production of honey and wax. Only a few ethnomedicinal studies on apitherapy have been published in Europe, highlighting hive products that are collected, sold, or used by beekeepers. The aim of this article is to provide a general overview of apitherapy practiced by beekeepers in different corners of Europe, namely Estonia, Ukraine, and Italy.

**Methods:**

We analyzed material from field studies conducted in three selected countries. From 2020 to 2024, we interviewed 17 beekeepers in each country. The average beekeeper interviewed was 55 years old, had approximately 45 beehives and approximately 22 years of experience, and did beekeeping as a part-time job. We also made observations at regional fairs and markets, as well as noted products originating from beekeeping in shops and pharmacies.

**Results:**

The most well-known and popular apitherapy products in all three countries were honey, pollen, propolis, and royal jelly. Due to the increasing market demand for health-promoting products, beekeepers have started to enhance their products by mixing beekeeping products into honey, making tinctures, performing bee sting treatments, etc. However, strict regulations prohibit beekeepers from labeling their products with health-promoting information. In addition, a completely new trend has emerged: apitherapy tourism. However, Italian beekeepers did not collect or use specific products made in Ukraine and Estonia, such as dead bee tincture, honeycomb moth larva tincture, and drone brood homogenates, and did not make honey moonshine.

**Conclusions:**

The development of apitherapy in Europe has depended on the development of beehive types, the advancement of beekeeping technology, and new knowledge about the health-giving properties of beekeeping products (promoted in the literature and by institutions). As beekeeping is closely related to market demand, apitherapy tourism has emerged as a completely new economic branch and apitherapy is becoming increasingly important in providing relief from mental health issues. However, this requires an entirely new approach from beekeepers and clients using apitherapy.

## Background

### Biocultural importance of the European honey bee

The European honey bee (*Apis mellifera* L.) is the most important insect species in human care in Europe. Its original distribution area included Europe, Africa, and the Near East. Its native distribution in Europe, which included several subspecies [1], covered all the continent with the exception of Iceland, the Faroe Islands, the Azores, and northern Scandinavia. Today, wild colonies of European honey bee are becoming rare across the European landscape [2]. In fact, it is still being determined whether the honey bee continues to occur in the wild, because of the introgression of managed and feral colonies [3, 4]. Requier et al. [2] argue that wild colonies still exist in protected areas, such as the biosphere reserve of the Swabian Alb, Germany, nesting in beech trees. Scholars have recently presented the case for restoring wild populations, for example of the northern European dark bee (*Apis mellifera mellifera* L.), by ‘rewilding’ free-living colonies for nature conservation purposes and as a reserve for future needs [5].

As the wild population of European bees is very limited, domesticated bees have become crucial in plant pollination [6]. However, labeling the European honey bee as domesticated is a little inaccurate. Despite human control over these bees, the habits of the species have not been affected, but rather are the same whether the bees are wild, feral, or kept by humans [7]. People in Europe have cared for honey bees since the Neolithic era when honey started to be collected. With European colonization of the globe starting in the fifteenth century, the European honey bee has spread to other continents and is now found wherever the ecological conditions for beekeeping exist [8]. However, pollinators are threatened by climate change, biodiversity loss, land degradation, and deforestation [9]. Besides their essential function in food and medicinal production, bees play a significant role in cultural, recreational, and emotional aspects, as sources of inspiration and traditional benefits for humans [9].

In Europe today, it is possible to make a living from only the sale of honey for beekeepers with at least 150–200 bee colonies. The general trend, for example in Estonia, is that most commercial honey will come from very large beekeeping companies in the future. Already today, there are about a dozen companies in Estonia that have more than 1000 hives (the largest has nearly 2,000) and they produce most of the honey on the market [10]. A similar trend is observed in Italy, where a small percentage of professional beekeepers (3.26%) manage over 34.45% of the hives, indicating a concentration of production among larger operators [11].

### Overview of the importance of beekeeping products and apitherapy in Europe before the period of commercialization

Bees have been important to people in Europe in many ways. Honey was the only sweetener available for a long time [12]. During the Bronze Age, beeswax played an important role in various manufacturing processes, including bronze casting. Later, wax was used to make candles, especially important for churches, as well as molds and seals [8]. Another important bee product is mead (also known as honey wine), which is mentioned in Norse mythology and is still produced in the Balkans, Sweden, and elsewhere. In the nineteenth century, the peasantry in southern Scandinavia kept bees in straw hives, mainly for the honey [1, 13, 14]. For example, in the latter half of the nineteenth century and up to the beginning of the twentieth century, it was common for primary school teachers to tend beehives. They could have quite a few hives and, as there was no teaching in the summer, they had time to tend hives. Honey and other products also provided necessary income for the teachers' households [15].

Honey was mentioned by ancient doctors as a medicine, for example, to treat wounds. Honey is also found in ancient pharmacopeias and was used as a laxative. In addition, honey continues to be used as an in-home remedy for throat complaints, bronchitis, and rhinitis, as well as for rashes [16]. As a medicine, bees wax has also been used for a long time. For instance, Dioscorides wrote that wax has a warming and softening effect. It was prescribed to dysentery sufferers, among other patients. Propolis, a sticky, resinous substance, has long been used for medicinal purposes and was mentioned in Nordic drug tariffs and pharmacopeias as early as the seventeenth and eighteenth centuries. However, it was mainly used externally, especially as a *cerata*. Both yellow and white wax are found in older European pharmacopeias and were used as protective and covering agents and were included as ingredients in *cerata*, ointments, and plasters [17]. Yellow wax was prescribed into the nineteenth century as a palliative for intestinal disorders. Even the bees themselves could be prescribed in folk medicine. During the late Middle Ages in Denmark, it was believed that a woman could not get pregnant if she ate bees. Rubbing a crushed bee against your teeth prevented toothache, according to a record from Denmark [14].

### The period of beekeeping commercialization

Ukrainian beekeeper Petro Ivanovich Prokopovych (1775–1850) is considered one of the founders of commercial beekeeping and the father of the modern hive because he invented the frame hive in 1814. This was a crucial innovation because previously bees were killed before extracting honey. What changed beekeeping more broadly, however, was the world's first hive with movable frames and an opening top, which was patented in 1852 by American beekeeper Lorenzo Lorraine Langstroth (1810–1895). Yet, it was American beekeeper Charles Dadant (1817–1902) who made the top opening frame hive widely known. He was fluent in several languages and greatly contributed to European beekeeping through his popular articles. Open-top frame hives of the Langstroth and Dadant types are still among the most widely used in Europe today. However, there is an increasing shift toward smaller-sized box hives, invented by American beekeeper Clayton Leon Farrar (1904–1970) [18].

The introduction of frame hives allowed for the collection of various hive products and better production of honey and wax. The commercial production of pollen, royal jelly, bee venom, and propolis hardly existed before the 1950s—as the technology for the industrial collection of pollen and royal jelly was only developed in the 1940s, and a commercial method of collecting bee venom in a way that did not kill the bees was only developed in the 1960s [8].

### The period of apitherapy commercialization

Bohemian doctor (based in Slovenia) Filip Terč (1844–1917) is considered the father of modern apitherapy in Europe. He started testing the use of bee venom to treat rheumatism in the 1870s, and his work inspired other European scientists such as Bodog Felix Beck (1868–1942), a Hungarian physician who published the book “Bee Venom Therapy” in New York in 1935 [19]. Beck's comprehensive book on bee venom treatment changed the general attitude worldwide. It is now considered the bible in the field and has been reprinted several times since the author's death, the last in 1997 [20].

In Eastern Europe, the usefulness of bee products for treating people and improving health began to be widely promoted by the USSR [21, 22] at the end of the 1950s. For example, in the Estonian Soviet Socialist Republic, several books, brochures, and articles began to appear at that time, when beekeepers were being taught how to collect and use bee venom [23, 24], royal jelly [25, 26], propolis [27] and pollen [23], as well as how to use honey as a remedy [28].

In addition to the propaganda of using beehive products to improve health, pharmaceutical factories in the USSR began to manufacture medicines from the products of Soviet state beekeepers. For example, in 1969, a 30% alcoholic solution of propolis started to be produced in Tallinn [29]. In the 1970s and 1980s, mass pollen grains collection began in the Baltic Soviet republics in large state apiaries, and this yield was sold to other constituent republics of the Soviet Union and the Socialist Federal Republic of Yugoslavia. Its production was considerably more profitable than the production of honey [30]. Some state apiaries, however, only specialized in collecting royal jelly, while others in collecting bee venom. These products were the most expensive and were sold at the Tallinn Pharmaceutical Plant. After the collapse of the USSR, the state-run purchase of these products disappeared [30]. Romania became the most important center of apitherapy in the 1980s, producing dozens of medicines. These products also had a major impact on the Soviet Union [31].

In Western Europe, apitherapy was not widespread until a few decades ago. For instance, the market for propolis, royal jelly, and pollen only started to develop in the 1990s, and a few years afterward, a few national associations of apitherapy were founded (the German one in 1999, the French one in 2008, and the Italian one in 2015). These associations mainly share apitherapy knowledge among physicians, beekeepers, and the general public. On top of the potential medicinal bee products including honey, pollen, royal jelly, propolis, and bee venom [32], some of these associations also promote apitherapy houses, or small wooden houses that provide a space for beehives and a separate space for people to enjoy the smell of hives (which they consider beneficial for the respiratory tract) and the buzzing of bees, which favors relaxation [33, 34].

### Objectives of this research

Very few ethnomedicinal studies on apitherapy have been published in Europe, highlighting hive products that are collected, sold, or used by beekeepers. For example, one conference abstract reported that 95% of beekeepers in their Lithuanian sample had used hive products to strengthen their health, especially the elderly and those with a limited number of apiaries [35]. Another Croatian conference abstract reported that 82% of the interviewed beekeepers used their bee products for preventive or curative purposes, and that they valued beekeeping as a means of relaxation, physical activity, and escape from everyday problems. It was also emphasized that beekeeping supports their social interaction [36]. In a study conducted in Ukraine, beekeepers indicated that beekeeping gave them satisfaction ("beekeeping as a hobby, rest, art, a tool for self-realization"), and that they could benefit from apitherapy ("traditional medicine uses bee products for the treatment of various diseases") [37]. A survey conducted among beekeepers in England showed that working with bees helped beekeepers overcome the stress of lockdown and restore mental balance during the COVID-19 pandemic [38]. In Germany, hive products like honey, propolis, pollen, and royal jelly were used by (elderly) beekeepers to strengthen their health or treat a health condition [39].

The aim of this article is to provide a general overview of apitherapy practiced by beekeepers in different corners of Europe, namely Estonia, Ukraine, and Italy. Our study has two guiding research questions:

a) How do beekeepers value the health properties of honey and other hive products in the three selected European contexts? How are these products used?

b) What is the push and pull forces for apitherapy in the three selected countries??

## Materials and methodology

### Categories of beekeepers and general regulations in Estonia, Ukraine, and Italy

In Estonia, general food hygiene requirements apply to the production and sale of honey and honey-related products. Small-scale production of honey has been regulated since 2006 [40]. The regulation states that the most lenient control rules apply to beekeeping enterprises with less than 15 bee colonies and that sell only pure honey. Beekeepers with more than 15 bee colonies or sell mixed honey (e.g., with propolis or pollen) should possess professional certificates, have special processing rooms, and adhere to stricter hygiene requirements.

On the basis of production, beekeepers in Estonia are divided into the following categories: hobby beekeepers who have ten or fewer bee colonies, of which there are nearly five thousand in Estonia; beekeepers with 10–24 beehives are considered small producers, of which there are nearly 500; beekeepers with 25–99 beehives are regarded as medium producers, there are approximately 150 in Estonia; beekeepers with 100–149 bee colonies are large producers, there are about 15 in Estonia; and professional beekeepers are those who have more than 150 beehives—those whose main income derives from the sale of honey and honey-related products, there are about 20 such producers in Estonia (see more 41). Some authors (e.g., 18) categorize beekeepers into only three categories: hobby beekeepers with up to 15 bee families; semi-professionals with 16–70 bee colonies for whom honey represents an additional income; and professional beekeepers with more than 70 bee colonies for whom beekeeping provides the majority of their income.

In Estonia, the beekeeper must report the number of bee colonies they possess to the state agricultural register both in the spring, reflecting how many bee colonies survived the winter, and in the autumn, indicating how many bee colonies are put into hibernation. The locations of registered beehives in nature are visible to everyone with precise coordinates and beekeeper contact information (see https://mesi.ee/). However, there are quite a few hobby beekeepers who do not meet this state requirement. Today, Estonian beekeepers are very active socially, and they are divided into dozens of beekeeping organizations, societies, and associations based on both regional affiliation and professional competence (see more [41]).

In Ukraine, beekeeping activities are regulated by several laws, the main of which “On beekeeping” [42] is dedicated specifically to this occupation, as well as indirect legislation concerning beekeeping such as the "On plant protection", "On plant life", "On safety and quality of food products", and other normative legal acts. The norms specified in “On beekeeping” [42] provide separate forms and measures for the protection of beekeeping. Sellers of both honey and bee products (honey, wax, pollen, bee bread, propolis, royal jelly, bee venom, drone brood homogenate) are regulated by sanitary rules. Regardless of the number of hives, each apiary must have a bee passport issued by the regional state veterinary service. This law also regulates which bee breeds can be kept in a certain area, in which natural communities the apiary should be placed, and the protection of nature and bees.

Ukraine is a country with a centuries-long tradition of beekeeping and even today it remains one of the leaders in the production and export of honey. Ukraine remains the 5th largest world exporter by volume (after China, Argentina, India, and Mexico). Ukraine is also among world's five largest honey manufacturers (according to FAO) and for over 10 years it has been the leader in honey production in Europe. During the first 6 months of 2024, Ukraine exported 40.6 thousand tons of honey. Currently, the National Standard DSTU No. 4497:2005 "Natural honey" is working in Ukraine, which provides only voluntary requirements for the labeling, production, and sale of honey within the territory of Ukraine. Starting in 2028 new requirements for the export of honey and its labeling are planned (based on EU regulations). At the Honey Forum 2024, experts reported that more than 60% of honey is supplied to consumers directly from beekeepers. Another 25% passes through unofficial trade channels, and only 15% of the honey consumed by Ukrainians is the product of industrial processing, which has all the necessary aspects of production and packaging and is sold officially with the payment of taxes (Eduard Krychfalushiy notes).

In Italy, beekeepers who sell honey and hive products directly to consumers must adhere to strict regulations to ensure product quality and safety. Processing facilities must be clean, humidity must be controlled (< 65%), and the temperature must remain between 10 °C and 30 °C. Food grade equipment must be used for production operations such as extracting, filtering, and packaging. The extracted honey should be dehumidified if necessary and filtered before decanting or bottling. The liquefaction of crystallized honey is permitted for short periods at temperatures of up to 40 °C. For retail packaging, hermetically sealed glass containers must be used, which must be clean, inspected, and labeled in accordance with legal requirements. The labels must clearly state the product name, country of origin, net weight, producer details, and the batch or expiry date. Similar regulations also apply to other hive products to ensure safety and traceability [43]. In Veneto*,* there are 7,200 registered beekeepers (as of September 2022), which corresponds to 10.5% of the total in Italy. The sector is predominantly made up of small beekeepers, 88% of whom manage fewer than 20 hives, while only 3.26% are large beekeepers, who manage collectively more than 34.45% of the 95,592 hives in the country. Most beekeepers (85%) are members of professional associations, but none of them are officially recognized as Producer Organizations, which represents a development opportunity for the sector [11].

### Fieldwork

For this research, we analyzed the material of three field studies outlined below and made observations at regional fairs and markets, as well as noted beehive products in shops and pharmacies. The material collected during fieldwork was analyzed using the context analysis method.

The fieldwork conducted across Estonia was part of a larger qualitative study conducted among beekeepers. The main topics that beekeepers were asked about included their knowledge of nature and observed changes in nature, which honey plants are common in the region, how they manage their beekeeping and the economy, bee and beehive pests, their social activities and membership in a professional association, the ethics of professional activity, their use of honey, what honey and other bee products they produce, and which apitherapy methods they promote and/or use themselves. At the end of the interview, participatory observation was also conducted in the apiary. The interviews lasted 1.5 to 3 h. This research represents the first study based on the given data. Interviews were conducted up to the saturation point. The main survey was conducted in July–August 2023, and the sample consisted of small and medium-sized beekeeping family enterprises (ten beekeepers). Their contact details were obtained from the websites of professional societies. In addition, participatory observation of one beekeeper was conducted during the open day of an apitherapy beekeeping enterprise (https://honeywolf.ee/et/) that included product presentation and information about services. Four hobby beekeepers who do not belong to professional societies were contacted through recommendations. In August 2024, an additional survey took place in one family business (two beekeepers). Therefore, the sample included a total of 17 beekeepers (including four women), with a mean age of 59 years (Table 1).

**Table 1:** An overview of the age, experience, and number of hives of the interviewed beekeepers (EE—Estonia, UA—Ukraine, IT—Italy)

	Minimum	Maximum	Mean
Age of beekeepers	27 (EE); 23 (UA); 21 (IT)	87 (EE); 80 (UA); 73 (IT)	59 (EE), 56 (UA), 50 (IT)
Number of hives	5 (EE); 2 (UA); 7 (IT)	200 (EE); 43 (UA); 200 (IT)	65 (EE), 16 (UA), 45 (IT)
Years of experience	5 (EE); 3 (UA); 3 (IT)	70 (EE); 40 (UA); 43 (IT)	27 (EE), 19 (UA), 19 (IT)

The SW Ukrainian fieldwork among beekeepers addressed their observations of changes in nature, honey production technology, how honey products are used both for food and to strengthen health, how professional knowledge is shared, what types of honey they produce, what products they use, what subsidies they have received for beekeeping, among other topics. This research is the first study based on the given data. Fieldwork in Ukraine was carried out in the summer of 2020. Due to regulations during the COVID-19 pandemic, the interviews were conducted via the telephone. Informed consent was obtained prior to the interviews. All interviews were recorded and then transcribed. All interviewees were given the freedom to withdraw the data they provided at any time. Through a previous project about beekeeping conducted by Chernivtsi University between 2017 and 2018 (UA 01/2017 "Study of honey bee colony losses in Ukraine and Austria: Joint analysis of the data and risk factors, informational support of beekeepers"), we received telephone contact details of 72 beekeepers living in Putyla district. We called all these numbers. If the phone was not answered the first time, we called again after a short time. It turned out that seven of the numbers were wrong numbers, as the person who answered the phone was not the individual whose contact information was given to us. Forty-six individuals refused to talk to us or did not respond at all to repeated calls. One respondent agreed to an interview but did not answer the phone when we called back at the appointed time. The interviews lasted from 1 to 2 h. For the majority of Ukrainian beekeepers, beekeeping and selling hive products was a hobby, not a primary source of income. A total of 17 beekeepers (including five women) living and working in the Putyla district were interviewed (Table 1).

The main fieldwork in NE Italy was carried out from March to June 2021 in the Veneto region, during which 13 beekeepers were interviewed. All contacts were obtained from the Regional Beekeepers Association of Veneto. The study was part of a larger survey of beekeepers, in which nearly twenty semi-structured questions were asked. These included what types of honey they produce, what hive products they produce, what environmental changes they have noticed, what are the impacts of environmental pollution on beekeeping, what parasites and diseases affect bees, what are the origins of the beekeepers' professional knowledge, and what are the motivations of beekeepers and challenges of their work. Most of the interviews took place in the beekeepers' own apiary, but due to COVID-19 restrictions, a few interviews were conducted via video conferencing. Additional fieldwork was conducted via video link in November 2024 when an additional four beekeepers were interviewed. Therefore, a total of 17 beekeepers (including two women) were interviewed (Table 1). Only six beekeepers had the needed documents to be considered professional beekeepers; all the others who did not have this registration could only engage in beekeeping as a hobby.

During all interviews we followed the ethical guidelines of the International Society of Ethnobiology [44]. The purpose of the study and what would be done with the data collected was explained to all participants. Only those participants who gave verbal (and in Estonia also written) consent were interviewed. All respondents participated in the survey voluntarily and could discontinue the interview at any time. No names or sensitive personal information was recorded. We could not ensure a gender balance in the survey because the work of a beekeeper is physically demanding, and far fewer women do it. Data were transcribed from the interview recordings, and audio files were subsequently deleted as agreed upon with the interviewees. All the interview transcripts were searched in order to identify beekeeping products used to improve the health of beekeepers or demanded by their customers according to their narratives.

## Results

The beekeepers in our sample produced a very diverse range of bee products to strengthen health or treat various diseases (Table 2).

**Table 2:** Beehive by-products and types of apitherapy activities mentioned by the interviewed beekeepers (EE—Estonia, UA—Ukraine, IT—Italy)

Health products named by beekeepers	Local name	EE *n* = 17	UA*n* = 17	IT *n* = 17
Honey—for eating*	mesi (EE), мeд (UA), miele (IT)	4	1	
Honey—for lubrication, sometimes salt is mixed and used in a sauna	saunamesi, mesi (EE), мeд (UA)	7	1	
Bee bread (perga)	suir (EE), пepгa (UA)	8	3	
Pollen grains*	õietolm (EE), пилoк (UA, polline (IT)	6	14	4
Honey mixed with pollen grains	mesi õietolmuga (EE)	1		
Propolis*	taruvaik, propolis (EE), propoli (IT), пpoпoлic (UA)	4	16	9
Propolis tincture	taruvaigutinktuur (EE)	6		
Honey mixed with propolis	mesi taruvaiguga (EE)	5		
Bee venom* (including a bee sting for the purpose of improving health)	mesilasmürk (EE), veleno d'api (IT), бджoли́нa oтpýтa (UA)	8		
Bee therapy, e.g., Calming effects of leisure bee watching, lingering in a special cabin on top of a beehive	лeжaки нa вyликax, aбo бyдинoчки нa вyликax (UA), mesilaste teraapia maja (EE)	6	3	
Wax in treatment creams	mesilasvaha kreem (EE)	1		
Wax soap	mesilasvaha seep (EE)	1		
Wax cup with honey	вicк, зaбpyc (UA), kärjekaanetis (EE)	3	1	
Honey-based moonshine	meepuskar (EE)	2		
Mead (honey wine)	mõdu (EE), idromele (IT)	1		1
Dead bees (tincture)	surnud mesilased, podmor [пoдмop] (EE), пiдмop (UA)	9	2	
Royal jelly*	mesilasema toitepiim (EE), pappa reale (IT), мaтoчнe мoлoчкo (UA)	4	3	3
Drone brood homogenate	тpyтнeвe мoлoчкo (UA), lesevaglad? (EE)	1	2	
Honey mixed with royal jelly	mesi mesilasema toitepiimaga (EE)	1		
Honeycomb moth larvae tincture	мoль (UA), kärjekoi tinktuur (EE)	1	1	

*Classic beekeeping products known as health products, such as honey, propolis, royal jelly, and bee venom, were known to all beekeepers for their health benefits. The numbers show how many of these by-products they have actually used for themselves or collected for sale

All the interviewed beekeepers were aware of the health properties of honey, and sometimes, they attributed their good health to the fact that they regularly eat honey. We were told, and observed, that honey is always on the beekeeper's table and is often added to morning tea and coffee. Beekeepers also consider beekeeping a therapeutic activity and a way to improve mental health (Box 1). In Estonia and Ukraine, tea with honey is drunk, especially for treating viral infections. One female beekeeper from Estonia also used honey in a so-called "body cleansing drink", which consisted of mineral water, lemon (or apple vinegar), and honey. She said that you can only drink this healthy beverage for two weeks at a time. The difference between men and women also derives from the fact that only female beekeepers mentioned treating children: a honey mixture containing cranberry, garlic, and lemon for common colds; and honey applied to the chest for bronchitis.

**Box 1:** Beekeepers reflect on their experience in their apiary

You are the master of your time; you live according to nature, like bees. You don't have to go somewhere behind the workbench at seven o'clock in the morning, in the dark, like in the middle of the night, and then it's dark again when you come home. This is abnormal. For me, it's living in the right cycle, according to the sun, let's say,—in the summer, when it's a long day, you're like a bee doing a long day. At eight in the morning, you go with a lunch sandwich and come back from the forest at nine in the evening. You are with nature, you are always in the fresh air, it is a luxury. [–-] Sometimes I sit there on a stump, I hear a cuckoo calling, a bee buzzing, the sun is shining—life is the best job in the world. (Estonian, male, 56 years old)
For me, the biggest therapy in working with bees is that, imagine you go to the apiary in the spring when the weather is calm, when the liverwort is blooming, and the air is full of fragrance around you. [–-] The flowers are blooming—you just kind of sit there and think…this is fantastic, all the buzz…what a beautiful thing to just be and watch the flowers and the bees fly—it's like an enjoyment in itself. All this together creates such a beautiful milieu—all your senses capture it—your eyes look, your ears hear this hum, your nose smells all these scents, with your hands you enjoy all the activities you do in the apiary…you think, if only people knew how beautiful it is here! (Estonian, male, 60 years old)

A female Ukrainian beekeeper treated her mastitis with a honey compress, following the advice of a doctor. To do this, she mixed honey and flour and then put this on the breast. In Estonia, it was popular among beekeepers to use honey when giving a massage and in the sauna to smear on the body. Coarse salt is also sometimes added to sauna honey in order to better exfoliate old skin. Although there is a lot of sauna honey with salt brought from Finland in Estonian stores, the beekeepers in our sample did not sell sauna honey (with salt), but they made it for their own personal use. One beekeeper gave away beekeeping waste from honey processing in small plastic boxes for free, so that people could apply it to the body in the sauna. We also observed this product ourselves; it was a foam that formed on the top of fresh honey after it had been extracted and strained into a large honey barrel. It is mostly made up of tiny pieces of wax and a mixture of honey.

An Estonian couple (87-year-old woman, 86-year-old man), professional beekeepers since Soviet times, were the only ones who remembered the state-run mass buying of pollen grains in the 1970s and 1980s. Their state apiary was one of the first and largest pollen producers at the time. They also collected a small amount of royal jelly and bee venom at that time. These products are no longer purchased by the state; and therefore, industrial quantities are not produced. None of our interviewees in Estonia, Ukraine, or Italy collected pure bee venom and clean royal jelly for sale today.

The beekeepers we interviewed were also aware of the health benefits of royal jelly. Royal jelly was sucked directly from the queen’s cell, either by family members or children. However, our interviewees emphasized that children should not consume more than one queen cell per day. The collected queen cells were stored in the freezer and, if necessary, defrosted. Only one Estonian beekeeper had once made honey with royal jelly for a particular customer and also had sold frozen queen cells.

Pollen grains are also rarely collected because it is difficult to sell them in large quantities domestically. In addition, special pollen drying boxes are expensive to purchase. Pollen grains are packaged into small packets and sold. To a small extent, pollen-mixed honey is now also made by adding finely crushed pollen to the honey and sold. Pollen grains were crushed in an electric grinder (e.g., a coffee grinder). Ukrainian beekeepers said that pollen helps with many ailments, such as arthritis and arthrosis, and so a tincture is also made from them.

**Fig. 1:**
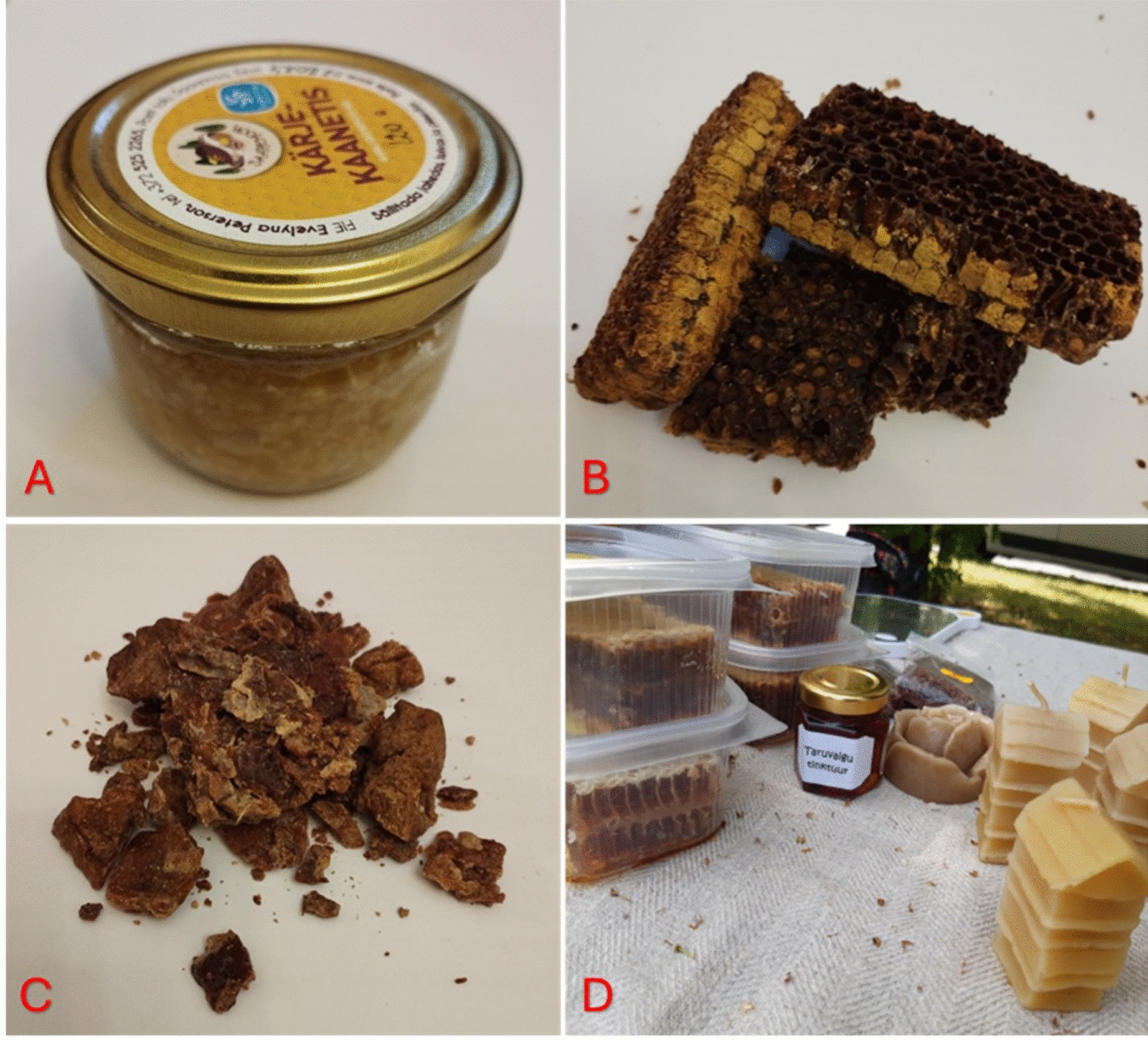
Products purchased from Estonian beekeepers: **A** wax caps with honey; **B** bee bread in pollen combs; **C** propolis; **D** homemade propolis tincture in a small glass jar (center of photo), honeycombs in plastic boxes (on the left), and homemade wax candles (on the right)—making candles from wax and selling them at fairs is one source of income for small-scale beekeepers. Credit: Raivo Kalle, 2023–2024

Eating bee bread (Fig. 1b) is popular among beekeepers in Estonia and Ukraine, and it is also produced for sale. For the production of small quantities of bee bread, household electric fruit dryers are used to dry the pieces of the pollen comb. In both Ukraine and Estonia, beekeepers ate bee bread to strengthen their health.

**Fig. 2:**
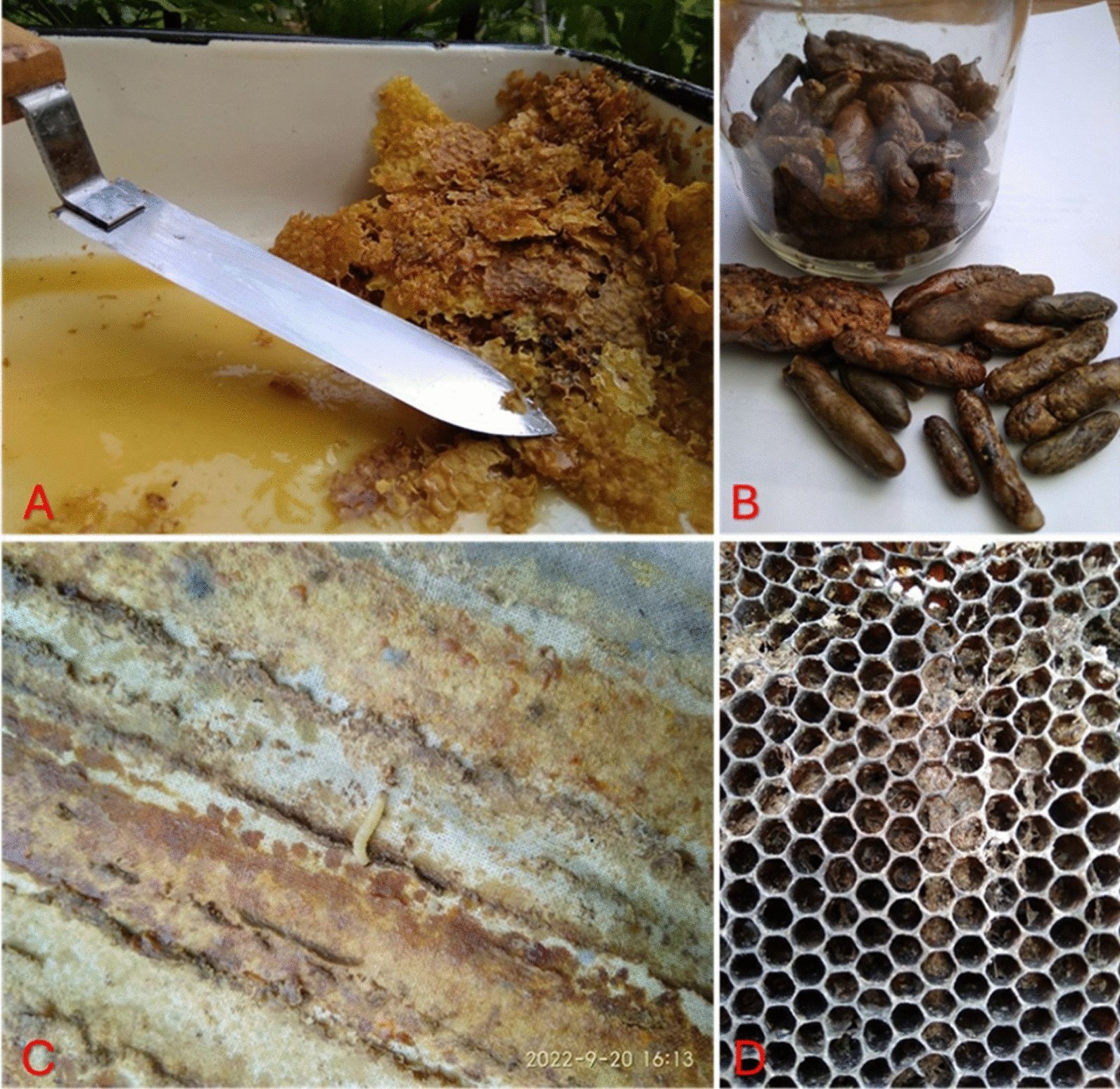
Hive products of Ukrainian beekeepers: **A** wax caps with honey; **B** propolis; **C** honeycomb moth (*Galleria mellonella* (L., 1758)) larva on the hive cover cloth; **D** wax frames with honeycomb moth damage. Credit: Serhij Stryamets, Lviv region, September 2022

As a new product, a tincture of honeycomb moth larvae is now made. This information derives from more recent apitherapy literature. In Estonia, only one apitherapy company produced this, and in Ukraine, small-scale beekeepers themselves also made such a tincture (Fig. 2c, d). In addition, as a new product, drone brood homogenate is now consumed to improve health. In Ukraine, drone brood homogenate is made into a so-called "vitamin bomb" at home, to which pollen and bee bread are also added.

Another new product is honey with wax caps—the latter of which are removed from the honeycomb before honey extraction (Fig. 2a). In Estonia, beekeepers have started selling this product in glass jars (Fig. 1a). It is eaten as a snack and children especially like to chew it—instead of chewing gum. Some Estonian beekeepers claimed that chewing this wax is healthy. In Ukraine, our interviewees also eat it when they have a sore throat.

Homemade creams and ointments made from wax are quite popular. They are sold, for example, at fairs and alternative medicine stores in Estonia. Beekeepers are aware of the use of such wax, but they do not make such creams themselves. Only one female beekeeper in Estonia practicing apitherapy made creams as well as wax soap for cosmetics.

In Estonia and Ukraine, honey processing residues and honey that is not suitable for sale (e.g., old honey from a bee colony that died during the winter) are fermented and made into moonshine at home. Although this is an illegal activity in Estonia (and Ukraine) and is not talked about publicly, two beekeepers dared to tell us about it. However, this is a fairly widespread activity. This homemade moonshine is used for disinfecting as well as for making compresses and tinctures, e.g., propolis tinctures.

Propolis (Figs. 1c and 2b) is a product known to all beekeepers as having antiseptic properties. All beekeepers have used it at least once, either on themselves or their family members, for the treatment of wounds and skin diseases. Ukrainian beekeepers also chew propolis for disinfecting the mouth and against the COVID-19 virus. To a small extent, propolis is also sold in small packages under a company brand. Making a propolis tincture with strong alcohol (Fig. 1d) at home is common among beekeepers in Estonia. It is used as a disinfectant and to treat inflammation, and older people also use it to treat varicose veins. Only a few beekeepers make this kind of tincture and sell it in small quantities. However, honey mixed with propolis (tincture) is very popular now in Estonia because it is a well-bought product.

Beekeepers were also quite aware of the healing effect of bee venom. Many Estonian beekeepers described themselves as already “being full” of bee venom, which maintains their health, and having received spontaneous bee stings during their work. Bee stings were also used to treat joint and back pain: The bee was captured and allowed to sting the painful area. Some Estonian beekeepers even had regular clients who came to them for bee acupuncture. Sometimes a so-called cure session is also performed, in which, for example, bee stings are applied 10 days in a row. As the occupational health risk of beekeepers is lower back pain, bee stings are primarily used to treat this complaint. At the same time, an apitherapy beekeeping company in Estonia treats a wide variety of diseases with bee stings. The beekeepers in the Italian sample have also all been spontaneously stung by bees, but they claimed to not practice bee acupuncture or any other form of apitherapy involving bee stings.

Making a therapeutic tincture from dead bees is also quite popular in Estonia, but in our sample, the beekeepers themselves did not use it but gave (or sold) dead bees to people who asked for them. Ukrainian beekeepers, however, have used a tincture of dead bees themselves. Dead bees are made into vodka and, in Ukraine, also homemade moonshine. This infusion is mostly applied to sore spots and very rarely it is ingested in small quantities (with a tablespoon) to treat internal diseases. Italian beekeepers do not use or sell dead bees for medicinal purposes. They collect the dead bees and send a sample to a laboratory to determine if the bees are free of disease. Bees that die in the hive during the winter are used as soil fertilizer in the garden.

As a completely new venture, beekeepers have started engaging in tourism for additional income. During our fieldwork, we visited four beekeepers in Estonia who had built special rooms or smaller houses for tourists, where they presented the history of beekeeping and the products of their company. Likewise, four Italian beekeepers in our sample organize visits to their apiaries for small groups to show their hives and provide information about beekeeping.

**Fig. 3:**
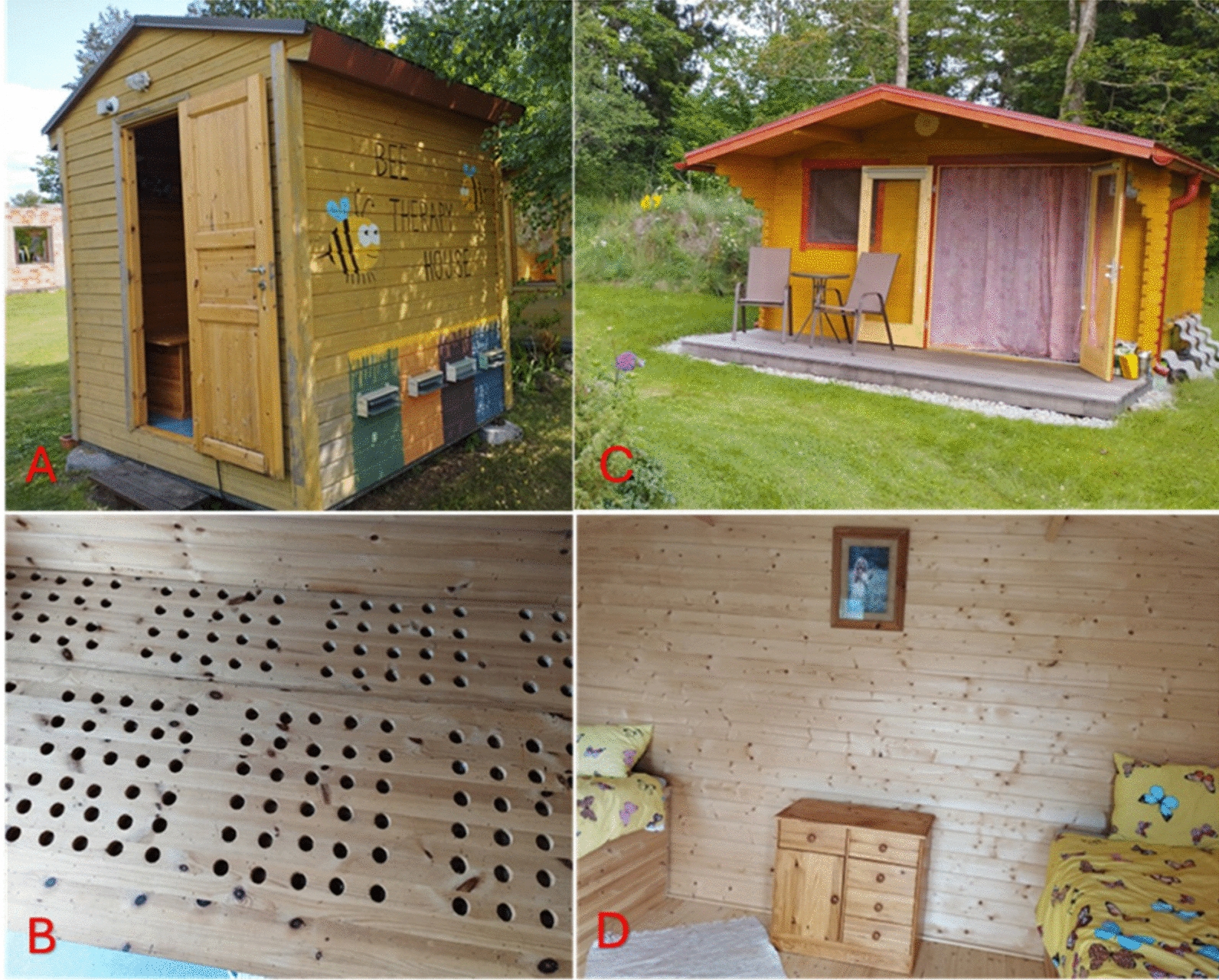
**A** two-person apitherapy house in Ida-Virumaa, exterior view, and **B** interior view showing the openings under the mattress of the bed, where bees can be seen. This “bee therapy house” was registered with the Estonian Patent Office (05.07.2016) and approved (15.02.2018) (Patent No. EE 01420 U1). **C** Two-bed apitherapy house in Saaremaa, exterior view, and **D** interior view. Credit: Raivo Kalle, 2023

Another new trend is apitherapy tourism, where visitors are offered relaxing, calming, and healing treatments in special houses, referred to as “beehives for tourist sleeping” or “bee therapy houses,” with a beehive under the bed (Fig. 3). Such buildings existed among both the Estonian and Ukrainian beekeepers we interviewed.

In Ukraine, propolis is sold in pharmacies as a tincture (*propolis tincture*) and is described as a “means for the treatment of wounds and ulcerative lesions.” In the description it is prescribed to treat microtraumas and superficial injuries of the skin and mucous membranes, as well as otitis, pharyngitis, tonsillitis, sinusitis, periodontitis.

Italian and Estonian stores sell a variety of propolis ointments, capsules, sprays, tinctures, etc., yet none of these items are labeled as health products. Buyers are expected to know for themselves what they should use these products for. The professional beekeepers in the Estonian sample have already had repeated encounters with the Agriculture and Food Board, and they have had to change the marketing of their honey products in accordance with government directions.

On the jar of honey, the official forbade me to say that honey "helps" [helps a person, with some ailment]. In addition, the official also checked my business website and started listing, it is not suitable, it is not suitable—that all this is misleading the consumer and must be taken away. […] I changed something on my website, but I put references behind the facts, which the official told me to take away. The final wording for the description of honey on my website remained, "Honey is a valuable, easily digestible food, it is healing and restorative," and the reference behind it. It took a long time for several officials to decide whether it was appropriate or not now. (Estonian, male, 56 years old)

The use of beekeeping products for treatment today is greatly influenced by online information. Estonian beekeepers said that Russian Federation doctors are particularly active in promoting treatment with bee products on the Internet. For example, when the promotion of dead bees appeared on Russian-language social media channels some time ago, the Russian-speaking people of Estonia began massively to search for and buy dead bees from Estonian beekeepers. Estonian beekeepers saw that there was a market for such a product and started collecting dead bees the following year as well. But then, suddenly, buyers' interest disappeared the following year, and there was no one to purchase the dead bees that were picked from the hive in the spring. This also illustrates the vulnerability of alternative courses: When the so-called fashion changes, former products are abandoned.

## Discussion

### Are apitherapy products new fashion trends?

Our results show that beekeepers produce and use various beekeeping products to strengthen their own health; however, there are also differences between the three countries. The difference between the Italian, Ukrainian, and Estonian data was that Italian beekeepers did not collect or use dead bees (tincture), honeycomb moth larva (tincture), and drone brood homogenates, nor did they make honey moonshine. Below we look at where and when these apitherapy products became known in Eastern Europe.

Honey moonshine, distilled at home from fermented honey wort, is quite well-known in Estonia (and also in Ukraine). In Ukraine, honey is also added to moonshine, which is called *medovuha*. Another drink is prepared with fermented honey, but beekeepers complained that it is difficult to make, and the recipe has been forgotten. However, as we were told, honey moonshine is not made in large quantities for sale, as the penalties for doing so are high. Rather, they make this drink either for themselves or for their friends.

Drone brood homogenate (3–7-day-old drone larvae) only became known in the 1980s. This product was patented by the Romanian beekeeper and beekeeping journalist Nicolae V. Ilieşiu (1913–1998), who started to promote this product in Europe under the name "Apilarnil" [45]. A lot of research has now been done on this product [46, 47] and it is still popular in Romania today, lyophilized or mixed with honey [48].

The honeycomb moth (*Galleria mellonella* (L., 1758)) is a dangerous parasite in beekeeping, and its activities can kill both bees in hives and bee colonies living in the wild. The larvae of this parasite feed on honey, honey in the comb, pollen in the comb, and sometimes on the bee's brood (only when food is scarce) [49]. Honeycomb moth larvae were introduced to Russian homeopathy by Sergey Alekseevich Mukhin (1905–1981) with his homeopathic medicine “Vita” developed in 1961. Research on honeycomb moth larvae as a medicinal product has continued in Russia since then [50] as well as in Ukraine [51].

Dead bees were probably first used in medicine by Dr. Naum Petrovich Yoirish (1905–1978), who was one of the best-known advocates of the medical use of bee venom (apitoxin therapy) and other beekeeping products in the USSR. Starting in 1938, he began researching the medical applications of honey and bee venom. After World War II, he continued conducting experiments and published over 200 articles and several influential books, such as “Bee Products and Their Use,” which has been translated into 30 languages, including French, Spanish, English, and Bengali. In his writings, he drew upon Russian-speaking sources and internationally recognized scientific literature. Yoirish developed techniques like honey bath and localized bee sting therapies, garnering recognition both in the USSR and abroad, including being named Honorary Beekeeper of Yugoslavia in 1975. He advocated the use of honey, bee venom, beeswax, propolis, pollen, and royal jelly [21]. Yoirish stated that in old folk medicine the decoction of dead bees was effectively used, and he noted that beekeepers in the Altay region were still using it in the 1970s. He was the first to say that bees that have died at the bottom of the hive before spring (*podmor*) are a great resource for the pharmaceutical industry [52]. The authors have not found any confirmation as to whether Yoirish's recommendation to start buying up dead bees from beekeepers was actually implemented anywhere in the USSR.

The application of a decoction of dead bees to alleviate toothache has been recorded in Lithuania. However, Lithuanian archival documents dating from 1886 to 1992 claim that “for epilepsy healing, the drinking of water with boiled dead bees (dead after the winter) was used” [53]. The use of an alcohol tincture with dead bees has also recently been recorded in Belarus for the treatment of cardiovascular disorders [54].

### New challenges in beekeeping and apitherapy tourism

Making a living solely from honey production is made difficult by high competition: the low market price of honey together with its large overproduction (for example, in Estonia). However, for the foreign market (Estonian), honey exports are hampered by the higher production price compared to southern areas [55]. Likewise, fake honey made from syrup, which is also extremely cheap, is crowding out more expensive real honey from the market. In addition, less and less honey is being bought per person in Europe. Therefore, small and medium-sized honey enterprises must find alternative sources of income. As a result, some Estonian beekeepers have now begun to valorize their honey, by mixing honey with propolis, pollen grains, or wax caps. These honeys are more expensive and sell better. Non-bee products are also mixed into honey, including freeze-dried berry powders, such as sea buckthorn, as well as freeze-dried spruce needle, black garlic, and quince powders among others. People are generally interested in buying these healthy products.

The practice of eating honey has disappeared among young people [10, 56]. To revive this habit among the youth, since 2007, Slovenian beekeepers have brought honey to kindergartens and primary schools every third Friday in November to promote a healthy and balanced diet [57]. This day of celebration of honey has now taken root in Estonia as well. In Italy, a recent survey revealed that the consumption of honey is closely related to its perceived healthiness and sustainability aspects [58]. These initiatives show that the health benefits are one component of honey's promotion.

However, beekeepers cannot inform clients about the health properties of honey as this is prohibited by law. As specified by Arno Bruder, a representative of the German Apitherapy Association [34], a beekeeper needs a license from the Medicines Board to share any health-related recommendations:

Many beekeepers harvest propolis and then process it into tinctures, creams and ointments and usually sell these products to their customers with health-related claims. However, if the beekeepers make health-related claims about the tinctures, creams, and ointments when selling propolis, a propolis product automatically becomes a medicinal product. In this case, pharmaceutical law applies. Arno Bruder [59].

Apitherapy tourism, a new economic branch, has also emerged. Some of the more active small and medium-sized beekeeping companies already receive additional income from providing apitherapy services, as they can no longer survive from selling honey alone. Sachuk et al. [60] pointed out that the popularity of apitherapy is increasing in Ukraine, and apitherapy tourism has the potential to become an important economic branch in some regions. The first Ukrainian beekeeper who build houses with beehives was Ivan Prysyazhnyj in 1988. He later modified this pavilion for bees and people, and in 2006 in Truskavets Resort the bee-house was built, where the promotion of apitherapy was carried out [61]. The first such Bee-Therapy House was patented in Estonia in 2018 (registered in 2016). Since our interviews with Italian beekeepers, who mentioned that such activities were not yet legal in 2021, more apitherapy houses *apiari del benessere* [33] have been built all over Italy.

Although the job of a professional beekeeper is physically demanding, as we were told, the mental satisfaction gained is one of the reasons for doing it. Considering today's increasing mental health problems, bee therapy may become the main source of income for some beekeepers in the future. The reason is that maintaining special bee therapy houses requires a different beekeeping technology than today's intensive beekeeping. The importance of bee therapy houses in beekeeping education is also demonstrated by the fact that such a house is located right next to the Estonian beekeeping vocational school (see [62]).

The future of apitherapy houses depends a lot on marketing and advertising, regarding how beekeepers can share their positive experiences and the extent to which they reach the media with their activities. We think that it would also help if the positive emotional stories of other beekeepers were collected and applied more generally; for example, how they enjoy nature while working and what emotions bees evoke in them (see Box 1). In our opinion, this is one part of apitherapy.

## Conclusion

The development of apitherapy in Europe has depended on the invention of different types of beehives, the advancement of beekeeping technology, and new knowledge about the health-giving properties of beekeeping products. Our research shows that beekeeping, like any business, is closely related to market demands. In Europe today, it is increasingly difficult for beekeepers to support themselves in the traditional way, only by selling honey. This explains why more and more small and medium-sized beekeeping companies have started to valorize their honey—by adding several healthy components. Such products originate from market demand, as more and more customers appreciate healthy and functional food. However, beekeepers are subject to strict regulations with regard to describing the healthiness of their products when selling them.

Although the most expensive apitherapy products are royal jelly and pure bee venom, none of our interviewees specialized in their production. Our sample of beekeepers from Estonia and Ukraine sold pollen, bee bread, and propolis in small quantities under their own brands. As a new product, beekeepers in Estonia have introduced the sale of wax caps with honey. Italian beekeepers did not collect or use dead bees, honeycomb moth larvae, or drone brood homogenate for treatment, nor did they make honey moonshine or practice bee sting therapy.

Apitherapy tourism has emerged as a completely new economic branch. For example, Ukrainian and Estonian beekeepers have adjusted to this new demand by building apitherapy houses. We conclude that apitherapy is becoming increasingly important in providing relief for mental health problems. However, this requires an entirely new approach for both the beekeeper and the client using apitherapy.

## Data Availability

No datasets were generated or analyzed during the current study.

## Abbreviations

SW: Southwestern
NE: Northeastern
EU: European Union
FAO: The Food and Agriculture Organization of the United Nations
EE: Estonia
UA: Ukraine
IT: Italy
DSTU: State Standards of Ukraine
